# Supplementary material for: Multi‐dataset identification of innovative feature genes and molecular mechanisms in keratoconus
Source: J Cell Mol Med. 2024 Sep 19;28(18):e70079. doi: 10.1111/jcmm.70079 (PMC11412914; doi:10.1111/jcmm.70079)
Supplement: Supplementary file 2 — Material S2. [file JCMM-28-e70079-s002.zip › 7.GSEA analysis/2.ATOH7/KEGG_PHENYLALANINE_METABOLISM.html]

Details for gene set KEGG\_PHENYLALANINE\_METABOLISM[GSEA]

|  || Dataset | ATOH7.group.cls#h\_versus\_l |
| Phenotype | group.cls#h\_versus\_l |
| Upregulated in class | l |
| GeneSet | KEGG\_PHENYLALANINE\_METABOLISM |
| Enrichment Score (ES) | -0.37304556 |
| Normalized Enrichment Score (NES) | -1.0228348 |
| Nominal p-value | 0.4392157 |
| FDR q-value | 1.0 |
| FWER p-Value | 1.0 |
Table: GSEA Results Summary

  

Fig 1: Enrichment plot: KEGG\_PHENYLALANINE\_METABOLISM      
 Profile of the Running ES Score & Positions of GeneSet Members on the Rank Ordered List

  

| SYMBOL | TITLE | RANK IN GENE LIST | RANK METRIC SCORE | RUNNING ES | CORE ENRICHMENT || 1 | HPD | na | 607 | 0.519 | 0.1543 | No |
| 2 | ALDH1A3 | na | 1890 | 0.353 | 0.2342 | No |
| 3 | AOC3 | na | 3743 | 0.247 | 0.2625 | No |
| 4 | ALDH3B2 | na | 7831 | 0.122 | 0.1852 | No |
| 5 | ALDH3A1 | na | 10357 | 0.076 | 0.1376 | No |
| 6 | IL4I1 | na | 11846 | 0.055 | 0.1128 | No |
| 7 | PRDX6 | na | 12513 | 0.046 | 0.1088 | No |
| 8 | MIF | na | 14343 | 0.027 | 0.0651 | No |
| 9 | GOT1 | na | 17581 | -0.000 | -0.0280 | No |
| 10 | MAOA | na | 19096 | -0.014 | -0.0671 | No |
| 11 | PAH | na | 21121 | -0.033 | -0.1145 | No |
| 12 | ALDH3B1 | na | 22158 | -0.045 | -0.1296 | No |
| 13 | GOT2 | na | 23507 | -0.062 | -0.1478 | No |
| 14 | DDC | na | 25136 | -0.088 | -0.1657 | No |
| 15 | MAOB | na | 32336 | -0.315 | -0.2687 | Yes |
| 16 | TAT | na | 33413 | -0.406 | -0.1652 | Yes |
| 17 | AOC2 | na | 34386 | -0.613 | 0.0099 | Yes |
Table: GSEA details [plain text format]

  

Fig 2: KEGG\_PHENYLALANINE\_METABOLISM      
 Blue-Pink O' Gram in the Space of the Analyzed GeneSet

  

Fig 3: KEGG\_PHENYLALANINE\_METABOLISM: Random ES distribution      
 Gene set null distribution of ES for **KEGG\_PHENYLALANINE\_METABOLISM**
